# Stress and Hypertension Among African American Female Family Caregivers of Persons Living With Alzheimer Disease and Related Dementias: Protocol for a Pilot Internet-Based Randomized Controlled Trial

**DOI:** 10.2196/66975

**Published:** 2025-03-27

**Authors:** Kathy D Wright, Ingrid K Richards Adams, Nathan P Helsabeck, Karen M Rose, Karen O Moss, Donya Nemati, Navia Palmer, Bohyun Kim, Sunita Pokhrel Bhattarai, Christopher Nguyen, Daniel Addison, Maryanna D Klatt

**Affiliations:** 1 The Ohio State University College of Nursing Columbus, OH United States; 2 College of Food, Agricultural, and Environmental Sciences The Ohio State University, College of Medicine Columbus, OH United States; 3 Office of Research The Ohio State University College of Nursing Columbus, OH United States; 4 Department of Psychiatry and Behavioral Health The Ohio State University College of Medicine Columbus, OH United States; 5 Center for Integrative Health The Ohio State University College of Medicine Columbus, OH United States

**Keywords:** African American women, high blood pressure, stress reactivity and resilience, caregiving, hypertension, stress, Alzheimer disease, dementia, lifestyle and healthy self-care behaviors

## Abstract

**Background:**

Caregivers of persons with Alzheimer disease and related dementias (ADRD) neglect their health, including by ignoring stress levels. African American women are vulnerable and susceptible to hypertension. Chronic caregiving stress and hypertension place them at high risk for cardiovascular disease. Addressing stress reactivity or resilience is vital in lessening their caregiving stress, enhancing their quality of life (QOL), and fostering healthy blood pressure (BP) self-care behaviors.

**Objective:**

This pilot study aims to investigate the feasibility and acceptability of implementing the Mindfulness in Motion (MIM) plus the Dietary Approaches to Stop Hypertension (DASH) intervention in this population and to evaluate its effect on ADRD caregivers’ stress and QOL. Additionally, it explores the mediation of stress reactivity or resilience between interventions and self-care behaviors.

**Methods:**

A small randomized controlled trial pilot study will recruit 28 African American or Black female caregivers aged 40 years diagnosed with hypertension and on an antihypertensive medication. Participants will be randomly assigned to either the MIM DASH or the Alzheimer’s Association caregiver training group (attention control). Trained facilitators will deliver both interventions over 8 weeks through 1-hour, group, internet-based sessions, via video or telephone. After completion, both groups will receive coaching calls over 9 months, beginning with 8 weekly calls followed by 4 monthly calls to encourage use of the educational materials. Primary outcome measures include feasibility (recruitment and retention) and acceptability (attendance). Secondary measures assess caregiver stress (Perceived Stress Scale), QOL, and self-care behaviors (Food Frequency Questionnaire and self-reported physical activity). Data collection occurs at baseline, 3 months, and 9 months. Quantitative data will be analyzed using descriptive statistics, CIs, and mediation models.

**Results:**

This study was approved by the institutional review board in April 2022 and funded in May 2022. The first data were collected in January 2023, and the last data were collected in September 2024. The completion of all aims’ data analysis is anticipated in spring 2025. The participants’ mean age was 62.4 (SD 7.98) years, with a mean baseline systolic BP of 128 (SD 19) mm Hg and diastolic BP of 79 (SD 10) mm Hg. Participants reported that MIM DASH was acceptable (at a mean score of 59.08, SD 7.38, compared to 60.83, SD 5.56 for caregiver training). Regarding feasibility, as reflected in attendance, MIM DASH participants had a mean attendance of 6.3 (SD 2.3) sessions, and the caregiver training group had 4.9 (SD 2.9) sessions.

**Conclusions:**

This study’s findings demonstrate the feasibility of conducting an internet-based intervention (MIM DASH) for African American women with hypertension who also care for families living with ADRD. These results will inform the design of a larger randomized controlled trial to evaluate the intervention’s efficacy and scalability further.

**Trial Registration:**

ClinicalTrials.gov NCT05721482; https://clinicaltrials.gov/study/NCT05721482

**International Registered Report Identifier (IRRID):**

DERR1-10.2196/66975

## Introduction

### Background

Family caregivers of individuals with Alzheimer disease and related dementias (ADRD) provided an estimated 18.4 billion hours of unpaid care, valued at nearly US $350 billion [1]. The prevalence of ADRD is higher among African American individuals, leading to a disproportionately more significant share of the family caregiving burden. Approximately one-third of these caregivers of individuals living with dementia acknowledge postponing or neglecting their health due to their caregiving role [1]. Given African American individuals’ heightened risk of ADRD, particularly vascular and mixed dementia, this problem disproportionately affects African American family caregivers [1].

Although African American caregivers generally express positive feelings about caregiving and report cultural justification for providing care [2,3], they nonetheless endure the adverse effects of chronic caregiver stress—increased cortisol levels, hypertension [4,5], and consequently, cardiovascular disease [6]. Compounding their risk, African American women face a heightened danger of uncontrolled hypertension and cardiovascular disease [7]. No demographic group is more at risk for the double jeopardy of caregiving stress and hypertension than African American women caring for a family member who has ADRD.

Hypertension is the leading cause of cardiovascular disease among African American women, affecting 56.7% of this group [8]. Numerous studies recommend lifestyle changes such as stress management, reducing sodium intake, increasing the intake of fruits and vegetables, weight management, and engagement in regular physical activity to manage hypertension [9,10]. Researchers have hypothesized that African American individuals face excess chronic stress that makes adopting healthy self-care behaviors difficult [11,12].

One of the underlying mechanisms behind adopting healthy self-care behaviors is stress reactivity or resilience—the body’s psychological and physiological response to stress [13]. Understanding these factors can contribute to more effective and sustainable behavior changes such as healthy eating, stress management, and physical activity.

Only a few studies have explored the stress and resilience associated with healthy self-care behaviors among African American caregivers. No research to date has used an interdisciplinary methodology to examine the intricate relationship between stress and resilience in this demographic. This protocol paper describes a mind-body intervention designed to address the complex interplay of stress and hypertension in African American caregivers.

### Purpose

This randomized controlled pilot study examines the feasibility and acceptability of Mindfulness in Motion (MIM) combined with Dietary Approaches to Stop Hypertension (DASH) to promote hypertension self-care in African American women who are family caregivers of people living with ARD.

MIM uses gentle yoga stretches and teaches mindful awareness skills, while the DASH program promotes a diet rich in vegetables, fruits, whole grains, and lean proteins [14,15]. These interventions combined provide a comprehensive strategy for stress management and hypertension reduction. The control group will receive the Alzheimer’s Association’s caregiver training [16-19].

### Study Aims

This study aims (1) to determine the feasibility and acceptability of MIM DASH and caregiver training for African American female caregivers with hypertension; (2) to compare the influence of MIM DASH on caregiver stress and quality of life (QOL) with the influence of caregiver training; and (3) to investigate the potential mediation effects of stress reactivity or resilience between each program and self-care behaviors.

### Theoretical Models

#### Of MIM

Developed in 2004, MIM was based upon the ideas of Urie Bronfenbrenner’s Socio-Ecological Model, which highlights the multilevel influences on behavior [20]. The Socio-Ecological Model has been extensively researched and is an effective way to structure multilevel interventions to aid in the long-term adoption and sustainability of programming [21]. MIM’s combination of dietary education and experiential mind or body practices aligns with the multilevel Socio-Ecological Model of change. MIM was first implemented in 2009 as a small, randomized controlled trial with 48 employees [22]. Guided by the question of “Is MIM effective?” the program was tested in various additional studies with medical center faculty, staff, residents, and patients [23-30]. These trials showed significant stress and burnout reduction results with improved resilience.

#### Of DASH

The 8-week DASH education uses a critical thinking model of behavior change and culturally appropriate approaches to emphasize eating vegetables, fruits, whole grains, fat-free or low-fat dairy choices, fish, poultry, beans, and nuts and using vegetable oils [31]. Additionally, it limits foods high in saturated fat, sugar-sweetened beverages, and sweets. Critical thinking provides individuals with the tools for reasoning, empowerment, problem-solving, and making sound decisions [16]. The DASH eating plan decreases cardiovascular outcomes, including subclinical injury and systematic inflammation biomarkers, and improves blood pressure (BP), especially among African American individuals [32]. The critical thinking approach allows individuals to analyze information and their behavior to make fully informed decisions in the best interest of African American individuals; it also provides opportunities for reflection.

#### MIM and DASH

MIM and DASH both address health behaviors but from different perspectives. MIM integrates mindfulness practices, while DASH emphasizes critical thinking and heart-healthy dietary choices. Together, they contribute to holistic well-being and improved health outcomes.

### Study Timeline

The participants will be tracked for 9 months in the 2-year funded trial. Data will be collected at baseline, at 3 months, and during a 9-month follow-up.

## Methods

### Design

**Figure 1:**
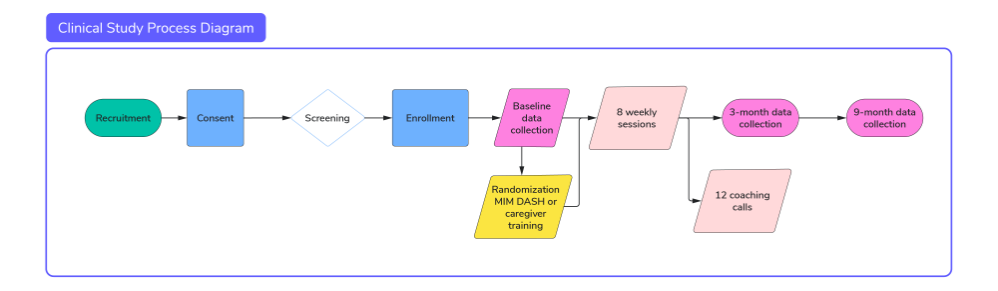
Flow Chart for the Study: The figure visually represents participants' enrolment and randomization into training or intervention programs, followed by their sessions and data collection phases.

In this randomized controlled trial, 28 community participants will be enrolled and randomly assigned to MIM DASH (n=14; intervention group) or caregiver training (n=14; attention-control group). This study’s team will use REDCap (Research Electronic Data Capture; Vanderbilt University) to deliver informed consent to prospective participants via an online check box. Figure 1 provides a flow chart for this study.

### Recruitment

Participants will be recruited from clinics, the African American Alzheimer’s and Wellness Association, the Central Ohio Alzheimer’s Association, local churches, the Research Match registry, and social media. Additionally, study flyers will be displayed in places potential participants are likely to frequent, such as libraries, beauty salons, health provider offices, and senior centers. To maintain confidentiality, the list of all potential participants will be securely kept separate from the documentation and tracking spreadsheet in the REDCap. Basic demographic information and reasons for refusal will be noted for eligible individuals who decline participation. Enrollment and screen failure data will be tracked using the National Institutes on Aging (NIA) Common Data Screening, and enrollment forms will be reported to the NIA once every month (NIA Clinical Research Operations & Management System) [33].

### Eligibility

Study participants will be adults aged 40 years and older who self-identify as Black or African American women and as family caregivers to someone living with ADRD. Inclusion criteria include the following: (1) have a diagnosis of hypertension that is treated with an antihypertensive medication; (2) have a score of two or greater on the eight-item Informant Interview to Differentiate Aging and Dementia [34]; (3) provide unpaid care for a person living with ADRD at least 10 hours per week and assist a person living with dementia with at least 1 instrumental activity of daily living (eg, bill paying, medication management, or transportation); (4) speak English; and (5) have access to a telecommunications device such as a desktop computer, laptop, tablet, smartphone, or telephone, to enable participation. Exclusion criteria include the following: (1) expect to move out of the area within nine months; (2) have a diagnosis of resistant hypertension (BP that remains above goal despite concurrent use of a diuretic or water pill and at least two other antihypertensive medications of different classes), or (3) are actively participating in a mindfulness or yoga program.

### Randomization

All caregivers who enroll in this study and complete the baseline assessment will be randomly assigned into either the intervention group (MIM DASH) or the attention-control group (caregiver training). The rationale for an attention-control condition is to strengthen this study’s design and minimize the chance that differences between the groups could be related to receiving social support from interacting with others [14]. Once one of this study’s staff members has completed the baseline data collection, the statistician will randomly assign this study’s participants using a randomization table generated from the statistical software in the REDCap database. Trial participants will be blinded to this study’s hypotheses.

### MIM DASH (Intervention Group) Delivery

A trained MIM facilitator and a dietitian will deliver the intervention in eight 1.5-hour (~45 minutes for MIM and ~45 minutes for DASH) sessions via Zoom (Zoom Video Communications, Inc) with telephone access. Participants will receive session materials such as PowerPoint (Microsoft Corp) slides to follow along by phone or the Zoom videoconferencing app. Table 1 provides a list of the topics.

**Table 1:** Weekly topics for MIM^a^ DASH^b^ (intervention group) and caregiver training (attention-control group).

Week	MIM DASH	Caregiver training
1	Introduction to MIM What African American individuals should know about hypertension and its consequences	Healthy brain and body
2	Mindful sleep Understanding blood pressure overview	10 warning signs
3	Vision of self Clearing up myths about hypertension	Dementia conservations
4	Mindful eating Basics of the DASH diet	Effective communication
5	Balance through movement Be a DASH detective: sodium is the culprit	Understanding behaviors
6	Sensation DASH: throughout your day—breakfast, lunch, and dinner	Safety and driving
7	Clarity and release DASH: when eating out	Physician’s visits
8	Staying grounded and moving forward DASH: diet is only part of the story	Money and legal

^a^MIM: Mindfulness in Motion.

^b^DASH: Dietary Approaches to Stop Hypertension.

Each MIM session will consist of mindfulness-related material—the somatic mind and body connection, relaxation, yoga, meditation, self-awareness, and bodily cues relating to emotional reactivity. Group interaction will center on sharing ideas toward effective practice and practical daily challenges to being mindful. Each class will begin with a prompt for participant contemplation during the session that references a unique weekly theme, which will be reiterated in the session materials. Then, the participants will be led through a body scan, gentle stretching, yoga, progressive relaxation, and mindful eating meditation, ending with formal meditation. Each participant will receive a new weekly link to online video mindfulness practice recordings. The workbook has a diary section to document study activities. The diary is personalized and retained by the participant. Participants will be instructed to perform mindfulness video meditations at least 5 times weekly and record the time in their diary.

The DASH portion focuses on education to increase intakes of vegetables, fruits, and whole grains and decrease intakes of fat, sodium, sugar-sweetened beverages, and sweets. The education includes adapting traditional “soul” food dishes to meet the DASH dietary guidelines. Participants will be provided practical tips on incorporating DASH into their daily lives. They will receive an individual MyPlate (United States Department of Agriculture Center for Nutrition Policy and Promotion) displaying serving sizes and food groups comprising a balanced meal and a home BP monitor. Principal investigator KDW will provide training on using the BP monitor and American Heart Association infographics and video [35,36]. Repetition of key concepts will be embedded throughout the sessions to increase critical thinking and problem-solving [17]. After completion of the 8-week sessions, the MIM DASH participants will receive 8 weekly and then 4 monthly coaching calls to review sessions and support the adoption of self-care behaviors. Our team has successfully delivered the MIM DASH intervention for older African American individuals with hypertension [14].

### Caregiver Training (Attention-Control Group)

A study team member trained by the Alzheimer’s Association will deliver the caregiver training. Participants in this group will attend eight 1.5-hour group lessons via Zoom for 8 weeks (Table 1). The training uses the Alzheimer’s Association caregiver topics listed in Table 1. The Alzheimer’s Association materials are based on the latest scientific evidence of dementia researchers and practitioners in partnership with various community-based, academic, and health care organizations [37].

As with the MIM DASH group, participants will receive educational materials to follow along using Zoom videoconferencing or phone. Participants will have group discussions and role-play using a case study scenario. After completing the 8-week sessions, participants will receive 8 weekly and then 4 monthly social calls to encourage them to maintain their involvement and enhance study retention for subsequent data collection [14].

### Fidelity

To maintain the fidelity of the intervention, a detailed protocol manual will be developed for MIM DASH and caregiver training. The MIM facilitators will be enrolled in 8 weekly 1-hour sessions as participants and attend a half-day workshop with return demonstrations. Before leading an MIM session independently, the facilitators will observe an 8-week MIM course and be the designated fidelity checker [38]. During each weekly session, the facilitator will show MIM educational and practice videos created by coinvestigator MDK. For the DASH component, the registered dietitian, also a coinvestigator IKRA, will develop a training plan and materials. Coinvestigator IKRA will deliver the DASH component and train others if needed. The materials for DASH will include a scripted facilitator manual. The principal investigator will also be trained to deliver MIM DASH as a backup facilitator. A study staff member will use a fidelity checklist for MIM DASH to observe the facilitators.

### Sample Size and Power Analysis

Our sample size of 28 participants (14 participants per arm) conforms to the recommended sample size rules of 20-30 for pilot studies, as there are diminishing returns for precision, particularly over sizes of 12 per group (“rule of 12s”) [39-41]. Further, a sample of 28 is considered a sufficient pilot sample to determine variance in a main trial powered to 80% to detect an effect of 0.4 [41]. In line with pilot studies, the sample size lacks the power to detect a small-to-medium effect size for aim 3. Therefore, our mediation analysis will not emphasize statistical significance but report point estimates, 95% CIs, and effect sizes.

### Measurements

Data collection measures consist of surveys, systolic and diastolic BP, the Pittsburgh Stress Battery, a Food Frequency Questionnaire, and the collection of a hair sample for cortisol analysis. Study staff were trained and observed before independently collecting data. Table 2 provides a data collection timetable, including the constructs, measures, and brief psychometrics provided in validation studies. These data will be collected in the treatment and attention-control groups at baseline, 3 months, and 9 months. Each study visit is estimated to take 2 hours. Maximum flexibility is critical to engage busy caregivers in research. Our team offers several options for data collection. Study visits may be conducted at the participant’s preferred time, day, and location. For example, participants may select the visits at their home, a dedicated research study office, or a private room at a local library. Study visits can take place outside of traditional office hours and workdays. The participant may have this study visit broken up into two 1-hour visits if needed. In these cases, the research staff will prioritize the collection of data that requires an in-person visit (eg, BP and hair collection) and offer to meet again in person or via telephone or Zoom to collect the remaining data.

**Table 2:** Data collection variables, measures, and psychometrics.

Construct and measures			Psychometrics						
			Baseline		3 mo		9 mo		Reliability and validity
**Pre-enrollment screening**									
	AD8^a^	✓						The area under the curve is 0.908 (95% CI 0.888-0.925)	
**Biologic**									
	Age in years	✓						N/A^b^	
	List of comorbidities	✓						N/A	
**Measure of health literacy**									
	Newest Vital Sign	✓						α=.74	
**Self-care behaviors**									
	Stress management practices survey part A	✓		✓		✓		α=.71	
	Healthy Eating Index: Block FFQ^c^	✓				✓		Test-retest reliability, *r*=0.59	
	DASH^d^ index: Calculated from the Block FFQ	✓				✓		The mean correlation coefficient between frequencies of intake of 55 foods assessed by 2 FFQ 12 months apart=0.57	
	Medication list and BP^e^ log	✓		✓		✓		C statistic=0.704	
	Krousel-Wood medication adherence	✓		✓		✓		C statistic=0.704	
	Systolic and diastolic BP	✓		✓		✓		κ=0.68	
**Stress reactivity or resilience**									
	Daily inventory of stressful events	✓		✓		✓		κ ranged from 0.66 to 0.95	
	Pittsburgh Stress Battery	✓		✓		✓		Heart rate α=.93; systolic BP α=.92; and diastolic BP α=.93	
**Stress or QOL^f^**									
	Perceived Stress Scale (caregiver stress)	✓		✓		✓		α=.83	
	Folkman 1 question regarding what is most stressful	✓		✓		✓		α=.83	
	Discrimination in the health care setting consists of 7 questions on a Likert scale to measure the stress of discrimination in the health care setting as a result of age and ethnicity	✓		✓		✓		α=.89	
	Hair cortisol (chronic stress proxy)	✓				✓		Correlation with 30-day saliva (*r*=0.42, *P*=.04)	
	WHO-5^g^ (QOL)	✓		✓		✓		Sensitivity 0.93; specificity 0.83	
**Depression-PHQ-9^h^**									
	PHQ-9 has 9 questions to evaluate mild, moderate, or severe depression	✓		✓		✓		PHQ-9 α=.90	
**Generalized anxiety disorders**									
	GAD-7^i^ is a 7-item instrument that is used to measure or assess the severity of generalized anxiety disorder	✓		✓		✓		GAD-7 α=.92	
**Revised memory and behavior checklist**									
	32-item checklist that assesses activities of daily living and problem behaviors in people living with ADRD^j^	✓		✓		✓		α=.84	
**Credibility**									
	The credibility scale has 5 questions			✓				α=.86	
**Acceptability**									
	Acceptability of participant preferences has 13 questions			✓				N/A	

^a^AD8: 8-item Informant Interview to Differentiate Aging and Dementia.

^b^N/A: not applicable.

^c^FFQ: Food Frequency Questionnaire.

^d^DASH: Dietary Approaches to Stop Hypertension.

^e^BP: blood pressure.

^f^QOL: quality of life.

^g^WHO-5: World Health Organization-5.

^h^PHQ-9: Patient Health Questionnaire-9.

^i^GAD-7: Generalized Anxiety Disorder Assessment.

^j^ADRD: Alzheimer disease and related dementias.

### Pre-Enrollment Screening

We will screen potential participants using the Informant Interview to Differentiate Aging and Dementia. The survey is a dementia screening interview that differentiates typical signs of aging from dementia [42]. The instrument contains 8 items to examine memory, orientation, judgment, and function. The instrument demonstrated strong discrimination in a validation study (area under the receiver operating characteristic [ROC] curve=0.834) [42]. A score of 2 or greater indicates that cognitive impairment is likely present.

### Biologic

Age in years and a self-reported list of comorbidities will be obtained at baseline.

### Measure of Health Literacy

At baseline, we will administer the 6-item Newest Vital Sign (Pfizer Inc) to assess functional health literacy [43]. The data collector will ask the participants to read a food label for a pint of ice cream. Then, participants will be asked questions regarding serving size, calories, carbohydrates, saturated fat, and food allergies. Scores range from 0 to 6, with lower scores indicating lower health literacy. Scores of less than 4 indicate the possibility of the respondent experiencing low literacy. The scale was initially validated in a sample of 492, including English- and Spanish-speaking adults residing in the United States. Our study will use the English version of Newest Vital Sign that has adequate internal consistency (α=.76) and excellent discrimination (area under the ROC curve=0.88) [4].

### Self-Care Behaviors

Four measures will be used to assess self-care. First, the stress management practices will be measured using the Measure of Current Status (MOCS) Part A [44]. MOCS is a list of 13 statements, such as “I am able to use muscle relaxation techniques to reduce any tension I experience,” to which participants respond using a Likert scale (0=I cannot do this at all to 4=I can do this extremely well). This scale also has 4 subfactors: relaxation, awareness of tension, having needs met, and coping confidence. Scores will be summed and range from 0 to 52, with higher scores indicating a more significant use of stress management strategies. Reported internal consistency (α) across MOCS Part A was .71, .77, .86, and .89 [44].

Second, the Block Food Frequency Questionnaire will be used as a validated measure with a food and beverage list that includes 127 items, plus supplementary questions to adjust fat, protein, carbohydrate, sugar, and whole grain content. The questionnaire also includes self-reported physical activity [45]. The questionnaire assesses the frequency with which the respondent generally consumes each food or beverage. It has 9 continuous responses ranging from “never” to “every day” for most foods. The Block Food Frequency Questionnaire provides a Healthy Eating Index score for diet quality (range 0-100) [46]. The DASH index will be calculated using the resulting data and a quintile system to score foods related to the DASH diet. All components are equally weighted. Vegetable, fruit (including fruit juice), nuts and legumes, and whole grains intake are scored from 1 (lowest quintile) to 5 (highest quintile). The overall DASH component scores range from 8 to 40.

Third, we will obtain a list of medications from participants. Finally, the Krousel-Wood Medication Adherence scale captures 4 domains of medication adherence behavior. It was developed to identify low adherence to medication refills in older adults [47]. The scale has reported adequate discrimination (area under the ROC curve=0.704). Scores range from 0 to 4, with a score of 1 or greater indicating lower adherence to medication refill behavior.

### Stress Reactivity or Resilience

Systolic and diastolic BP is measured 3 times, starting with a 5-minute rest [35]. Stress resilience and reactivity will be assessed using 2 measures. First, the Daily Inventory of Stressful Events was identified from the Science of Behavioral Change research network list of instruments recommended to assess stress reactivity or resilience**.** The instrument is a semistructured interview in which participants report whether specific stressful events had occurred within the past 24 hours. This instrument categorizes scores for each reported stressful event by examining (1) content classification of the stressor, for example, work overload, argument over housework, or traffic problem; (2) who was the focus of the event; (3) threat type experienced (eg, loss, disappointment, or frustration); (4) objective and subjective severity of stressors; and (5) primary appraisals (eg, areas of life that were at risk because of the stressor). Scores range from 0 to 27. This instrument will be distributed and the data will be collected via an automated text link from the Mosio app. Research staff will call participants who prefer not to use Mosio to deliver the inventory daily to gather the data. Second, the Pittsburgh Stress Battery is a test of stress reactivity. It measures BP response during stressful tasks. The tasks include the Stroop color matching test, mirror tracing, and mental mathematics. For example, during the mental math task, participants will be given 3 trials of fundamental arithmetic problems lasting 1 minute per trial. Participants who score 60% or greater will advance to the random-medium level; those who do not will repeat the easy level. Before the third trial, the data collector will place the BP cuff on the participant’s left arm unless contraindicated. At the 30-second mark of the third math trial, the data collector will use an automatic BP machine (clinician grade) to measure systolic BP, diastolic BP, and heart rate. CN is a neuropsychologist who will assist in the interpretation of data. The Pittsburgh Stress Battery was identified as a valid measure of stress reactivity by the Science of Behavior Change Research Network [48].

### Stress or QOL

We will use the Perceived Stress Scale, which has 10 items rated on a Likert scale with a reference range of 0-40 regarding stress over the past month, with higher scores indicating higher stress [49]. The scale was validated across 3 samples, 2 made up of college students and 1 from a smoking cessation program. Internal consistency across the 3 samples was 0.84, 0.85, and 0.86. Further, the scale correlated well with measures of stress symptoms (correlations ranged from 0.52 to 0.76), and the measure is appropriate for multiple applications. The Everyday Discrimination Stress in the Healthcare Setting Survey is a 7-item scale adapted for use in a medical setting from the Everyday Discrimination Scale [50]. The survey questions load to a single factor [51]. Using this scale, we will examine the respondents’ experiences and frequency of race-based mistreatment while accessing health care. The scale has a reported internal consistency of α=.89 and a test-retest reliability of 0.58. Further, the scale correlates well with the Krieger Experiences of Discrimination (*r*=0.51), a measure of societal discrimination.

Hair cortisol will be used as a proxy for chronic stress. To collect the hair samples, approximately 25-75 mg of hair (approximate width of shoelace tip when bunched) will be cut from the posterior vertex region of the scalp as close to the scalp as possible. The posterior vertex has the lowest variation in cortisol levels, making it the preferred area for sampling. To prep for assay, the hair sample is cut, washed twice with isopropanol, and dried over 1-3 days. A total of 10-75 mg of hair is placed into a microcentrifuge tube, minced, and then ground in a Retsch 400 Mill. A total of 1.1 mL of high-performance liquid chromatography–grade methanol is added to the ground sample and incubated for 18-24 hours at room temperature with constant agitation. The tubes are centrifuged at 5000 g for 5 minutes at room temperature to pellet the powdered hair. The entire amount (~1 mL) of supernatant is transferred to a clean microcentrifuge tube, and the methanol is removed by evaporation using a stream of air for 6-8 hours at room temperature. The cortisol extract is reconstituted in 100 μL of Salimetric immunoassay cortisol analysis diluent buffer. Samples are assayed in duplicate inter- and intra-assay coefficients of calculated variation. Hair cortisol levels are expressed in hair as picogram per milligram and generally logged due to skewed distributions as needed. Participants will be surveyed on corticosteroid use, as these medications may suppress cortisol levels, and hair care practices, such as frequency of washing, chemical treatments, and hair product use [52]. Hair samples will be collected at baseline and 9 months.

We will use the World Health Organization-5, a short questionnaire consisting of 5 Likert scale statements of well-being over the past 2 weeks [53]. Scores range from 0 to 25, with higher scores indicating greater well-being. The measure is adapted from more extended versions (10 items and 28 items) and is approved for use in diverse populations by the World Health Organization [53].

### Depression and Anxiety

The Patient Health Questionnaire (PHQ-9) is a widely used measure of depression symptoms and severity. The questionnaire’s 9 items describe conditions that the respondent may be experiencing and how frequently they experience those specific conditions. Each item is scored on a scale of 0-3, with 0=not at all and 3=nearly every day. Items are summed with total ranges of 0-27; scores of 5-9 are mild depression, 10-14 are moderate depression, 15-19 are moderately severe depression, and 20 are severe depression. The measure was initially validated by administering the questionnaire to 6000 patients in 15 clinics [54]. The PHQ-9 demonstrated 88% specificity and 88% sensitivity for a score ≥10. The Generalized Anxiety Disorder Assessment (GAD-7) is based on 7 items scored from 0 to 3. Like the PHQ-9, the GAD-7 asks respondents about the frequency of feelings and behaviors, with 0=not at all and 3=nearly every day. The score is then summed up with totals ranging from 0 to 21, with cutoff scores for mild, moderate, and severe anxiety symptoms being 5, 10, and 15, respectively. The GAD-7 was tested in a sample of 1184 patients and demonstrated strong reliability with both internal consistency (α=.92) and test-retest (0.83) [55].

### Memory and Behavior

The Revised Memory and Behavior Problems Checklist assesses the psychological comorbidities of the caregiver and the health status of the person living with ADRD [34]. It consists of 24 items that evaluate activities of daily living and problematic behaviors in people living with ADRD. Factor analysis has 3 distinct subscales related to memory, depression, and disruptive behavior. Scores range from 0 to 96, with higher marks indicating more behavioral problems in the care recipient. Overall, the measure demonstrated good internal consistency (α=.84), with subscale internal consistency ranging from 0.67 to 0.89.

### Feasibility and Acceptability

We will track the feasibility of recruitment by counting the number of participants screened per month, the number who are eligible, the number enrolled per month, the average time delay from screening to enrollment, and the completion of data collection [56]. We will track treatment-specific preference ratings (pre- and postintervention) for acceptability. The participants will complete the Acceptability of Participant Preferences 13-item Likert-type survey ranging from 1 (strongly disagree) to 5 (strongly agree). Interventionists will keep detailed intervention session records describing participant responses to the intervention. For the MIM DASH group, we will collect data regarding the usage of self-care equipment (home BP monitor and MyPlate) from follow-up coaching calls. We will use a credibility scale for both groups regarding the expectation of benefits of this study. The Credibility Scale (α=.86) measures attitudes toward the treatment condition and the participants’ expectation of benefit once the treatment has been explained [57,58]. The scale consists of 5 questions rated 0 (not at all confident) to 10 (very confident). Higher scores, up to 45, will indicate greater credibility of the treatment condition. This will also aid in determining participants’ willingness to be randomized for future studies.

### Data Analysis Plan

To assess data quality, we will check biweekly data for completeness, accuracy, timeliness, and consistency. These checks will be completed by examining frequencies and descriptive statistics, summarizing participant characteristics, and examining variable distributions. Once identified, data anomalies will be fully investigated, and remediation strategies will be considered as appropriate.

We acknowledge potential confounders, such as health literacy and problem behaviors that the care recipient exhibits, that could influence participation in the intervention and outcomes. As this study’s sample size is small, we are limited in the number of covariates that can be used in the analysis. Therefore, we will control for the participant’s health literacy and the participant’s report of problem behaviors of the care recipient.

To address aim 1, determining the feasibility and acceptability of MIM DASH and caregiver training for African American female caregivers with hypertension, we will conduct descriptive data analyses to report findings related to feasibility and acceptability. These analyses will include a summary of participant background characteristics, including age, educational attainment, relationship to the individuals they care for, and baseline health measures. Descriptive analysis will include frequency counts and percentages for categorical variables and mean, median, range, and SD for continuous variables.

Next, we will use descriptive statistics to examine the feasibility and acceptability of the MIM DASH intervention and caregiver training, including (1) the approach-to-enrollment ratio, (2) the proportion of participants that complete all 8 sessions of the assigned intervention arm and attention control arm, (3) frequency of completion of the follow-up calls, and (4) proportion of sessions that the interventionist deviates from the delivery of the intervention protocol. Further, we will report the mean rating of the MIM DASH intervention from participants and the proportion of participants rating the MIM DASH intervention positively (4 or above). We will report point estimates along with 95% CIs to indicate the precision of these estimates. Based on our previous experience enrolling African American participants in studies, feasibility will be defined by (1) enrolling at least 40% of potentially eligible patients and (2) completing at least 75% of the assigned intervention. Acceptability will be determined by an average rating of 4 or higher and at least 80% of participants rating the intervention positively.

To address aim 2, comparing the influence of MIM DASH on caregiver stress and QOL with the influence of caregiver training, we will conduct an intention-to-treat (ITT) analysis. Thus, all outcomes for enrolled participants will be included regardless of the intervention dosage. The Perceived Stress Scale will be used to measure caregiver stress, and the cortisol level extracted from hair will be a proxy measure of chronic stress. The World Health Organization-5 will be used to measure QOL. The data will be collected in person and documented in REDCap at baseline and 3 and 9 months after the intervention. Descriptive statistics, including mean and SD, will be used to summarize each primary and secondary outcome. We will also report mean differences, 95% CIs, and effect sizes for both within- and between-group differences at each visit. The fixed effect of MIM DASH intervention will be examined by mixed-effects models with measurements nested within persons.

To address aim 3, investigating the potential mediation effects of stress reactivity or resilience between each program and self-care behaviors, we will conduct a mediation analysis with self-care as the outcome (y), participation in MIM DASH as the predictor (x), and stress resilience or reactivity as the mediator (m). We hypothesize that heightened stress reactivity will diminish the relationship (between x and y). In contrast, heightened stress resilience will enhance the relationship between MIM DASH (x) and adopting self-care behaviors (y). To quantify these variables, we will use the Daily Inventory of Stressful Events and the Pittsburgh Stress Battery to measure stress reactivity or resilience. Self-care behaviors will be calculated using the Stress Management Practices survey and the DASH eating index by the Block Food Frequency Questionnaire. The data will be collected in person and by online surveys at baseline and 3 and 9 months after the intervention. We will report descriptive statistics for each of the measures. Finally, we will conduct a mediation analysis to examine if stress resilience or reactivity explains a significant amount of the relationship between MIM DASH participation and self-care behavior. The estimated effects of the mediation and 95% CIs will be reported.

### Ethical Considerations

This study has received ethical approval from the Ohio State University Behavioral and Social Sciences institutional review board (IRB; approval 2022B0031) and conforms to the World Medical Association’s Declaration of Helsinki requirements. All participants were provided written informed consent before their inclusion.

This study will use descriptive data through surveys and objective measures that include computer tasks while measuring BP, heart rate, and hair samples for cortisol level. The research has minimal risk, as the Ohio State University Behavioral and Social Sciences IRB deemed. There may be no direct benefit to the participant. However, their participation may provide data that will provide insight into ways African American women can reduce their BP and improve their overall health. Autonomy and rights will be given to participants, including the right of refusal at any time, without penalty or loss of benefits to which they are otherwise entitled. If a participant is a student or employee at Ohio State, their decision will not affect their grades or employment status.

The consent and study staff will also describe the use of Mosio for 2-way communication via text messaging. The restricted website associated with Mosio will contain the participant’s cell phone and study ID numbers, which the designated study staff member assigns. The participants can choose not to use the Mosio system at any time. Participants will be informed that there are no costs related to participation. They will not incur any additional charges related to text messages to and from Mosio over and above what they normally have.

All participants will receive the BP categories flyer from the American Heart Association and be instructed to consult their provider if they have a systolic BP higher than 180 mm Hg or a diastolic BP higher than 120 mm Hg. The participant will be instructed to seek immediate assistance or contact their provider. The data monitoring plan is in place and consists of a physician, nurse scientist, and a biostatistician who will review data and any unexpected events at least once per year. Participants will receive US $50 after completing each data collection point, including the home BP monitor, for a total of US $150.

Study staff will be carefully trained to protect participant confidentiality. They will work with participants to devise a plan for contacting them by phone, email, text messaging, or some other means determined by the participant and decide if messages may be left. Participants will be assigned a study ID number, and a master log will be created associating participant names and study numbers. The master log will be stored separately from the College of Nursing’s secure server. Only the primary investigator and designated study staff will have access to the master log, which will be destroyed at the end of this study. Study staff’s access to study data will be determined based on the relevance to their responsibilities. Only aggregate data will be reported for the dissemination of study findings. Duo Mobile authentication and Zscaler cloud security are used to access REDCap and the College of Nursing secure server.

To mitigate the risk that participants violate the privacy and confidentiality of others in their group sessions, we will ask that those participants (and their family or support person) refrain from discussing with other participants outside the group. Likewise, they will be asked not to acknowledge meeting if they encounter each other elsewhere. These steps are not foolproof, and participants will be informed of the associated risks at the time of consent.

## Results

**Figure 2:**
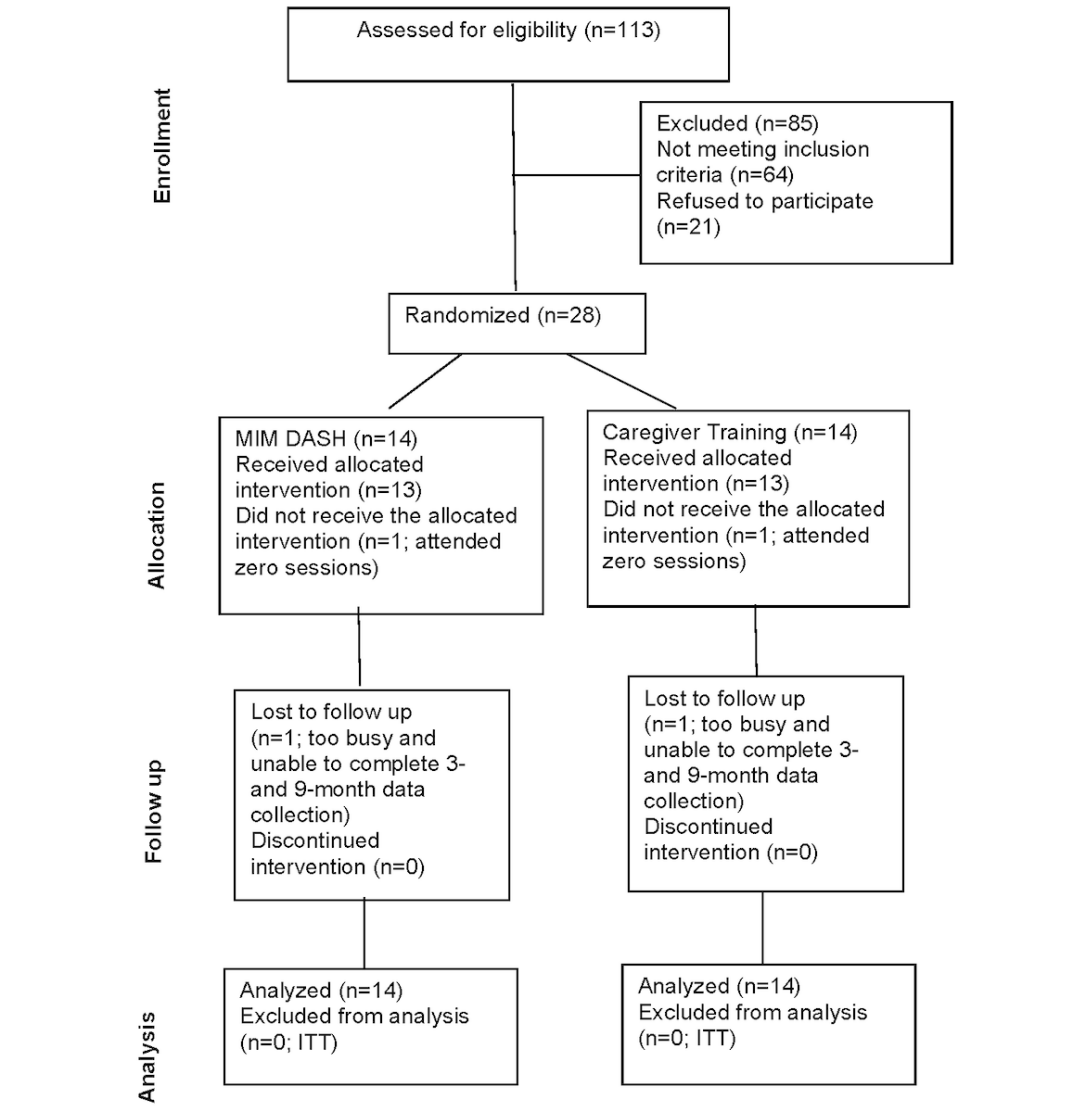
CONSORT flow diagram illustrating participant progression through the eligibility, allocation, follow-up, and final analysis stages of the clinical trial. CONSORT: Consolidated Standards of Reporting Trials; DASH: Dietary Approaches to Stop Hypertension; ITT: intention-to-treat analysis; MIM: Mindfulness in Motion.

The study was approved by the IRB in April 2022 and funded in May 2022. The first data were collected in January 2023, and the last data were collected in September 2024. Participants were recruited beginning in February 2023. Intervention delivery commenced in April 2023 for the first cohort and November 2023 for the second cohort. Completion of data analysis for all aims is anticipated in spring 2025. A CONSORT (Consolidated Standards of Reporting Trials) flow diagram is presented in Figure 2.

Of the 28 individuals enrolled, 82% (n=23) and 92% (n=26) completed the 3-month and 9-month data collection, respectively. Two participants were lost to follow-up. The mean age of the participants was 62.4 (SD 7.98) years. Eighteen participants were included in the first cohort, and 10 were included in the second cohort. 

The participants had a mean systolic BP of 128 (SD 19) mm Hg and diastolic BP of 79 (SD 10) mm Hg. The Newest Vital Sign (health literacy) screen had a mean score of 4.08 (SD 4.5), with a reference range of 0-6 where lower scores indicating lower health literacy. and The Perceived Stress Scale had a score of 12.75 (SD 6.7), with a reference range of 0-30 where higher scores indicating greater stress. Most participants reported that MIM DASH was acceptable (at a mean score of 59.08, SD 7.38, compared to 60.83, SD 5.56 for caregiver training). Regarding feasibility, as reflected in attendance, MIM DASH participants had a mean attendance of 6.3 (SD 2.3) sessions, and the caregiver training group had 4.9 (SD 2.9) sessions.

## Discussion

### Principal Findings

This paper presents a protocol to deliver a culturally responsive mind-body intervention (MIM DASH) specifically designed for African American women who have hypertension and are family caregivers of individuals living with ADRD. We hypothesize that the MIM DASH intervention will be feasible and acceptable, reduce stress, and improve QOL for African American female caregivers. Additionally, we explore behavioral change mechanisms, stress reactivity, and stress resilience that may explain how self-care is adopted or not, which can inform future studies. We hypothesize that lower stress reactivity and higher stress resilience will result in higher adoption of self-care behaviors. Although numerous studies support self-care behaviors (eg, mindfulness practice, healthy eating, and physical activity) to prevent cardiovascular disease—a complication of uncontrolled hypertension [59]—gaps remain in targeting interventions to address the complex interplay of caregiving stress and adoption of healthy self-care for African American female family caregivers of individuals living with ADRD. The findings from this study may contribute to developing and disseminating culturally responsive interventions to improve self-care behaviors and augment the treatment of hypertension and other cardiovascular disease risk factors.

Our principal finding thus far—that MIM DASH is feasible and acceptable—is similar to other studies that have used mindfulness as an intervention for stress and to promote healthy dietary habits [60-62]. Unlike previous interventions that lack cultural responsiveness or sufficient representation of African American participants, MIM DASH focuses on African American caregivers. This culturally responsive intervention integrates evidence-based approaches such as DASH and mindfulness, offering 8 weekly sessions focusing on healthy eating and stress reduction. The intervention also builds upon prior research but addresses critical gaps in cultural relevance and participant diversity. For example, noncaregiver studies on cardiovascular disease risk reduction, such as those promoting physical activity and healthy eating, often exclude culturally responsive strategies or include remarkably few African American participants, reducing their applicability to African American caregivers [59,60,63]. MIM DASH addresses these limitations by offering a potentially scalable, tailored approach that acknowledges this population’s cultural and systemic barriers.

### Limitations of This Study

This study will examine the feasibility and acceptability of the MIM DASH intervention in African American female caregivers of individuals living with ADRD. However, this protocol may have some limitations. The small sample size is a notable limitation, which may restrict the generalizability of the findings. Re-evaluation of inclusion criteria, including only those with a diagnosis of hypertension and those on an antihypertensive basis, may be too restrictive, making this study less generalizable. The multiple measures for data collection and the use of the Pittsburgh Stress Battery as measures of stress and resilience may increase the risk of burden and missing data. Although the Pittsburgh Stress Battery provides a standardized means of cardiovascular reactions to acute stress, it is limited in assessing chronic stressors. The limitations of using the Pittsburgh Stress Battery inventory include time to administer the test, challenges of internet connectivity to access the mirror test, and the cost of materials for administering the test. Other measures, such as heart rate variability, would burden participants and data collectors less [48]. Additionally, other theoretical mechanisms of behavior change, such as self-regulation (temporal discounting of immediate small rewards over larger future rewards) and interpersonal and social processes (social interactions), could be used as the facilitators of behavior change. Given that the MIM DASH intervention was delivered in a group setting and includes elements of self-regulation through mindfulness practice, future studies may consider exploring other, less costly mechanisms of behavior change.

Future studies with more extensive and diverse samples will be necessary to validate and scale the intervention. Furthermore, while this study aims to evaluate feasibility and acceptability, long-term outcomes such as sustained behavior change and clinical improvements in hypertension management remain unexplored.

### Future Directions

The findings of this study will be disseminated to this study’s participants in the form of a brief report or newsletter that will be submitted for approval to the Ohio State University IRB. Additionally, presentations will be given to the local African American community, churches, and Alzheimer’s disease support groups. The findings will be disseminated to the scientific community using multiple strategies, such as presentations at the Gerontological Society of America, the Alzheimer’s Association International Conference, and nursing and interprofessional peer-reviewed journals. By disseminating our study findings via traditional (researchers) and nontraditional (community), we will contribute to the science of behavior change and ADRD research for African American caregivers with hypertension.

To prepare for scalability, we will evaluate the delivery of the intervention from the facilitators’ perspective to determine what worked well and what needs to be refined. We are refining the training materials and facilitator fidelity monitoring for a larger trial. Data from the pilot feasibility study will help to inform the sample size for efficacy testing. To scale up, we will include additional measures of self-care management and behavioral change, such as actigraphy. We will use a framework such as Reach, Effectiveness, Adoption, Implementation, and Maintenance (RE-AIM), which provides a guide for evaluating the reach, effectiveness, adoption, implementation, and maintenance within the objectives of a larger randomized controlled trial [62]. If efficacy is successful, then using such a framework as RE-AIM would position the work for real-world implementation. Funding for the efficacy trial will be sought through grants from the National Institute on Aging or nonprofit organizations such as the American Heart Association, Alzheimer’s Association, or Bright Focus.

### Conclusion

The burden on African American dementia caregivers is expected to rise alongside the aging US population. Addressing the health of these caregivers is paramount to sustaining effective care for individuals with ADRD. The MIM DASH intervention represents a step toward providing culturally responsive hypertension self-care strategies for African American caregivers. This protocol sets the stage for improved caregiver health and contributes to the growing body of research on tailored interventions that address health disparities in minority populations.

## Appendix

Multimedia Appendix 1 Peer review reports by the MESH - Biobehavioral Mechanisms of Emotion, Stress and Health Study Section (National Institutes of Health, USA).

## Abbreviations

ADRD: Alzheimer disease and related dementias
BP: blood pressure
CONSORT: Consolidated Standards of Reporting Trials
DASH: Dietary Approaches to Stop Hypertension
GAD-7: Generalized Anxiety Disorder Assessment
IRB: institutional review board
ITT: intention-to-treat analysis
MIM: Mindfulness in Motion
MOCS: Measure of Current Status
NIA: National Institutes on Aging
PHQ-9: Patient Health Questionnaire-9
QOL: quality of life
RE-AIM: Reach, Effectiveness, Adoption, Implementation, and Maintenance
REDCap: Research Electronic Data Capture
ROC: receiver operating characteristic

## Data Availability

The datasets generated and analyzed during this study are not publicly available but are available from the corresponding author upon reasonable request. Data sharing will comply with ethical guidelines and any applicable regulatory requirements.
