# Supplementary material for: Stress and Hypertension Among African American Female Family Caregivers of Persons Living With Alzheimer Disease and Related Dementias: Protocol for a Pilot Internet-Based Randomized Controlled Trial
Source: JMIR Res Protoc. 2025 Mar 27;14:e66975. doi: 10.2196/66975 (PMC11986388; doi:10.2196/66975)
Supplement: Multimedia Appendix 1 [file resprot_v14i1e66975_app1.pdf]

SUMMARY STATEMENT
(Privileged Communication)

PROGRAM CONTACT:
Dr. Janine Simmons
301-443-6652
simmonsj@mail.nih.gov

Release Date: 11/22/2021
Revised Date:

Application Number: 1 R21 AG077069-01

Principal Investigator

WRIGHT, KATHY DENISE

Applicant Organization: OHIO STATE UNIVERSITY

Review Group: MESH
Biobehavioral Mechanisms of Emotion, Stress and Health Study Section

Meeting Date: 10/25/2021
Council: JAN 2022
Requested Start: 04/01/2022
RFA/PA: PA20-194
PCC: 2BPDIJS

Project Title: Addressing the double jeopardy of stress and hypertension among African American female caregivers of persons living with Alzheimer's disease and related dementias
SRG Action: Impact Score:32 Percentile:15 +
Next Steps: Visit https://grants.nih.gov/grants/next_steps.htm
Human Subjects: 30-Human subjects involved - Certified, no SRG concerns
Animal Subjects: 10-No live vertebrate animals involved for competing appl.
Gender: 2A-Only women, scientifically acceptable
Minority: 2A-Only minorities, scientifically acceptable
Age: 3U-No children included, scientifically unacceptable

Project Year	Direct Costs Requested	Estimated Total Cost
1	150,000	236,250
2	125,000	196,875
TOTAL	275,000	433,125

ADMINISTRATIVE BUDGET NOTE: The budget shown is the requested budget and has not been adjusted to reflect any recommendations made by reviewers. If an award is planned, the costs will be calculated by Institute grants management staff based on the recommendations outlined below in the COMMITTEE BUDGET RECOMMENDATIONS section.

1R21AG077069-01 Wright, Kathy

INCLUSION ACROSS THE LIFESPAN UNACCEPTABLE

RESUME AND SUMMARY OF DISCUSSION: This application proposes a two-group RCT of African American (AA) female caregivers 40 years of age and older with hypertension. Investigators will examine the feasibility and acceptability of the Mindfulness in Motion (MIM) and Dietary Approaches to Stop Hypertension (DASH) diet as well as assess the preliminary impact on caregiver stress and quality of life, while examining the potential mediation effects of stress reactivity and stress resilience between the intervention and self-care behaviors. If successful, findings could inform a larger trial assessing the same behavioral intervention. The committee discussed several strengths in the application, including a PI with foundational and culturally appropriate research expertise, a strong team of investigators with expertise in self-care interventions, chronic stress, tailored nutrition strategies, and biostatistics, the innovative combination of the mindfulness program with a nutritional strategy, and structural equivalence between the intervention and control arms. The panel also discussed several limitations, including insufficient detail on the qualifications/credentials of the instructors leading the MIM intervention, the absence of a community advisory board, the potential for spillover of interventions between the arms, and the absence of assessments of factors that could impact stress and uptake of intervention including health literacy, psychological comorbidity, ADL status, and stage of dementia. Overall, the panel believed this project could have moderate-to-high impact on our understanding of the role of healthy behaviors in self-care interventions to reduce racial health disparities in caregivers.

DESCRIPTION (provided by applicant): No demographic group is more at risk for the double jeopardy of caregiving stress and hypertension than African American women caring for a family member with Alzheimer’s disease and related dementias (ADRD). Both situations lead to reduced quality of life and cardiovascular disease—a complication of uncontrolled hypertension. Maintaining the health of these caregivers is critical to support the well-being of the care recipients. Although some multi-component interventions have addressed ADRD caregiver's stress and quality of life, gaps remain in targeting interventions to address the complexity of chronic caregiving stress and hypertension self-care in African American women. This pilot study builds on our earlier work which showed that stress, blood pressure knowledge, and complex diet information deficits all interfered with older African American women’s hypertension self-care. Lifestyle changes (stress management, reducing sodium, eating fruits/vegetables, and physical activity) are effective in managing hypertension. Our Stage I pilot study is based on the scientific rationale that we can promote these lifestyle changes by addressing stress reactivity/stress resilience, the psychological and physiological response of the body to stress, as the underlying mechanism to facilitate behavioral change. In this way we can improve health outcomes (caregiver stress, quality of life, cardiovascular disease risk). A small-scale two-group randomized controlled (RCT) pilot study of 28 African American female caregivers, age 40 and older with hypertension, will be conducted. We will determine the feasibility and acceptability of Mindfulness in Motion (MIM) plus the Dietary Approaches to Stop Hypertension (DASH). Participants will be randomized to either the MIM DASH intervention or the Alzheimer’s Association Caregiver Training (attention control) in 8 weekly, 1-hour group sessions via telehealth (video and telephone access). After completion of the intervention, both groups will receive four bi-monthly follow-up calls over the 12 months. To our knowledge, this is the first study that a) systematically employs one of the Science of Behavioral Change key mechanisms underlying successful adoption of health behaviors—stress reactivity/stress resilience and b) focuses solely on African American female caregivers of people living with dementia. The aims are: a) determine the feasibility and acceptability of MIM DASH and Caregiver Training for African American female caregivers with hypertension; b) explore the impact of MIM DASH as compared to Caregiving Training on caregiver stress and quality of life; and c) investigate the potential mediation effects of stress reactivity/stress resilience between MIM DASH or Caregiver Training and self-care behaviors. This pilot will make a substantive contribution to the science of behavior change by identifying basic mechanisms, in the adoption of healthy behaviors that can be

used to implement self-care interventions to reduce health disparities in African Americans. Study findings will inform the infrastructure for a larger trial.

PUBLIC HEALTH RELEVANCE: This study will test a behavioral intervention (MIM DASH) to promote hypertension self-care in African American women caregivers of people living with Alzheimer's disease and other related dementias. Findings from the pilot study will inform the infrastructure for a larger trial.

CRITIQUE 1

Significance: 2
Investigator(s): 2
Innovation: 3
Approach: 3
Environment: 2

Overall Impact: This R21 proposal plan includes a two-group randomized controlled (RCT) pilot study of 28 African American female caregivers (age 40 and older with hypertension) to examine the feasibility and acceptability of Mindfulness in Motion (MIM) plus the Dietary Approaches to Stop Hypertension (DASH). Participants will be randomized to either the MIM DASH intervention or the Alzheimer's Association Caregiver Training (attention control) in eight weekly, one-hour group sessions via telehealth (video and telephone access). After completion of the intervention, both groups will receive four bi-monthly follow-up calls over the 12 months. The study will test the preliminary impact of MIM DASH as compared to Caregiving Training on caregiver stress and quality of life, and the potential mediation effects of stress reactivity/stress resilience between MIM DASH or Caregiver Training and self-care behaviors. Participants will be randomized upon completion of baseline data. Measures will be collected at baseline, six months, and 12 months. An intent-to-treat analysis will be used. The study is intended to inform the infrastructure for a larger trial. The investigative team is very strong and complementary in its experience and expertise. The research environment provides outstanding resources, and the PI has established strong connections with relevant stakeholders. The combined mindfulness and nutritional strategy is innovative, and the structural equivalence between intervention arms is a strength. The measures, intervention fidelity plan, and analytic plan are adequate. A weakness is the omission of measures known to impact caregiver health, health behaviors, and response to behavioral interventions.

1. Significance:

Strengths

- The research focuses on the critical, unexamined interface between caregiving stress and hypertension in African American women.
- The literature presented offers the scientific premise substantiating the above problem, and the need for culturally sensitive interventions, and the hypotheses that MIM DASH addresses stress reactivity/stress resilience (the psychological and physiological response of the body to stress) as the underlying mechanism to facilitate behavioral change.
- The proposed study will address research gaps by conducting preliminary testing of a self-care intervention that focuses on mind-body integration to promote self-care, and the mechanistic action of stress reactivity/stress resilience, in African American female caregivers.

Weaknesses

- None.

2. Investigator(s):

Strengths

- The PI is well-prepared with very good foundational, culturally informed research and clinical experience to conduct the proposed research.
- The investigative team is strong, with the diverse expertise needed to conduct the study, including self-care interventions and chronic stress in African Americans, self-care interventions and chronic stress, tailored nutrition strategies, and biostatistics. Other experts represent cardiology and neuropsychology.

Weaknesses

- None.

3. Innovation:

Strengths

- Combining mindfulness and healthy eating to address two health adverse outcomes - stress and hypertension - is innovative.
- Examining the mechanistic action of stress reactivity/stress resilience in the intervention is innovative.
- The use of the Information-Motivation-Behavioral model is innovative.

Weaknesses

- There is limited information on how the innovation will be extended in the future RCT and on how results from this study will be foundational to the RCT.

4. Approach:

Strengths

- The intervention arms are structurally equivalent.
- The researchers have a strong, culturally appropriate recruitment plan, informed by past experience, and supported with community relationships. The retention plan is robust.
- Solid measurement plan includes feasibility and acceptability of the MIM DASH intervention, and the use of biologic, self-care, and stress reactivity/resilience/quality of life measures.
- Fidelity evaluation includes validation training of research staff and interventionist, as well as delivery, receipt, and enactment of the intervention.
- The analytic plan is appropriate with mixed effects models, mediation analyses, and multiple imputation for missing data.

Weaknesses

- Other factors are not examined that can potentially impact uptake and response to behavioral interventions, including health literacy and psychological morbidity.

5. Environment:

Strengths

- The research environment at the Ohio State University (OSU) is strong.
- The PI has described the resources that will be used in the project and prior activities including research and clinical collaborations within the extensive network at the university.
- Letters of support are provided from the African American Alzheimer's and Wellness Association, OSU Center for Cognitive and Memory Disorders, Wexner Medical Center Geriatric Clinical Outpatient Clinics, and OSU Federally Qualified Health Center.

Weaknesses

- None.

Study Timeline:

Strengths

- Adequate timeline, appropriate for the study.

Weaknesses

- None.

Protections for Human Subjects:

Acceptable Risks and/or Adequate Protections

- Comprehensive and complete.

Data and Safety Monitoring Plan (Applicable for Clinical Trials Only): Acceptable

- Adequate plan.

Inclusion Plans:

- Sex/Gender: Distribution justified scientifically
- Race/Ethnicity: Distribution justified scientifically
- Inclusion/Exclusion Based on Age: Distribution justified scientifically
- Adequate plan.

Vertebrate Animals:

Not Applicable (No Vertebrate Animals)

Biohazards:

Not Applicable (No Biohazards)

Resource Sharing Plans:

Acceptable

Budget and Period of Support:

Recommend as Requested

CRITIQUE 2

Significance: 2
Investigator(s): 1
Innovation: 4
Approach: 5
Environment: 2

Overall Impact: African American women experience double jeopardy -- caregiving stress and hypertension -- when caring for a family member with Alzheimer's disease or other related dementias (ADRD). In this study, the PI seeks to understand the extent to which African American female caregivers can change their lifestyles to manage hypertension risk effectively by improving lifestyle risk factors including stress reactivity, reducing sodium intake, improving access to fruits/vegetables, and increasing physical activity in a small RCT (N = 28 female ADRD caregivers). The long-term impact of hypertension remains one of the only health behaviors that can appear to reasonably cause an individual benefit in terms of own ADRD risk. The fact that spouses of ADRD patients are often at elevated risk of ADRD makes this study even more impactful. The study is being proposed by a KL2 scholar and promising early investigator. The MIM-DASH program proposed is a comprehensive multimodal intervention and appears to appropriately target hypertension risk in Black Americans at higher risk of ADRD. MIM-DASH includes a mindfulness task that incorporates the usual mindfulness breath work alongside some movement-based work including a yoga and an eating meditation delivered electronically. The investigators hope to test the view that improving mindfulness will reduce stress reactivity and improve resilience, thereby improving self-care behaviors. They hope that the intervention will be feasible and acceptable to the population of interest. The work will contribute to and collaborate with the Ohio CTSA program. The DASH component seeks to help participants manage their diets via work with a registered dietician. The PI's work engaging with the community is a critical strength. While there are significant strengths, some consideration of APOE4 genotype may be useful. Aim 3 is underpowered and mediation analysis usually requires more statistical power, but this also highlights how this is an interdependent aim. There is not a substantial amount of information indicating that the population size is sufficient to ensure the potential to recruit a huge number of caregivers in a new study. The program has a nice potential pathway to follow-up studies, otherwise.

1. Significance:

Strengths

- The long-term impact of hypertension remains one of the only health behaviors that can appear to reasonably cause an individual benefit in terms of own ADRD risk. The goal of this study is to take individuals who are at greater risk because of multiple overlapping risk factors in order to intervene directly with those individuals. This insight is actually quite novel and important.
- Finding ways to improve ADRD risk is a critically important topic.
- Hypertension risk is higher in Blacks, as is ADRD risk. Additionally, the excess ADRD risk is not really well understood and therefore this makes this particular intervention particularly important.

Weaknesses

- There is no discussion of the fact that spouses of ADRD patients are often at elevated risk of ADRD as compared to other individuals, likely due in part to clusters of poorer risk behaviors. This makes this study more important, so it would be good to discuss the impact of dyadic risk here.

- While the significance of the problem is important, the history in behavioral change has been fairly clear that it rarely works. It would be useful to clarify why this particular intervention is likely to improve behaviors where others have failed.
- Reliance on education-based interventions is somewhat detracting for me. The mindfulness component improves the protocol but may be less acceptable.
- A minor issue, but the citations do not appear to match the references, so it was difficult to examine the extent to which the proposal had already been completed.

2. Investigator(s):

Strengths

- The PI is capable of accessing and engaging with this difficult-to-reach community, and therefore in a unique position to reasonably accomplish the study aims.
- Nursing staff and nursing researchers are particularly critical for managing behavioral tasks such as these ones, and are well respected in the community; therefore, they may be seen as critical sources of information.
- The integration of a dietician in this study is a strength.
- The statistician and data manager are critical to the study's feasibility.

Weaknesses

- It may be worth bringing in community advisors to help with informing and selling this work.

3. Innovation:

Strengths

- The MIM-DASH program is a nice multimodal intervention.

Weaknesses

- There is some lack of clarity as to whether the intervention is novel or is being repeated in this population after having already been completed in other circumstances.

4. Approach:

Strengths

- The recruitment/retention strategy is appropriate and suggests that the authors have considered the difficulties with recruitment in the area.
- Incorporating a measure of hair cortisol is novel and may be important.

Weaknesses

- The appropriate sample size for an initial phase-I trial, as per the intervention scientists when power is unknown, is often accepted to be n=30 split evenly between arms. The use of 28 participants is odd but this is a minor concern.
- Some consideration of APOE4 genotype may be useful – APOE4 carriers are at higher risk of ADRD and also for hypertension. Since they are relatively uncommon (if more common in Blacks), it may be important to consider the effects of APOE4 on the study's outcomes.

- Intent to treat is appropriate, but what is the likelihood that people stop using the intervention and switch their group status? Since it is information-based, is there a concern that these older women are likely to share their experiences outside of the intervention's observation space and cause spillover? There is no discussion of this, but it's very likely in small populations like this who are often linked together based on their role as caregivers. The Alzheimer's Association has operations to do stuff just like this.
- Aim 3 is underpowered, but that is expected with exploratory aims. Mediation analyses usually require more power than other analyses. Also, it requires that an effect is present, which is not yet evident so this aim is interdependent and will fail if other aims also fail.

5. Environment:

Strengths

- The institution has the resources to accomplish this task.
- The local population appears to be large enough to support an initial intervention.
- The environment seems ideal for this program in many critical ways.

Weaknesses

- There is not a substantial amount of information indicating that the population size is sufficient to ensure the potential to recruit this many caregivers. As the program scales up, this population may be limiting, and multisite trials may be required.

Study Timeline:

Strengths

- Seems fine.

Weaknesses

- None noted by reviewer.

Protections for Human Subjects:

Acceptable Risks and/or Adequate Protections

- Data collected in a rigorous and acceptable way.

Data and Safety Monitoring Plan (Applicable for Clinical Trials Only): Not Applicable (No Clinical Trials)

Inclusion Plans:

- Sex/Gender: Distribution justified scientifically
- Race/Ethnicity: Distribution justified scientifically
- Inclusion/Exclusion Based on Age: Distribution justified scientifically

Vertebrate Animals:

Not Applicable (No Vertebrate Animals)

Biohazards:

Not Applicable (No Biohazards)

Resource Sharing Plans:

Acceptable

Budget and Period of Support:

Recommend as Requested:

CRITIQUE 3

Significance: 4
Investigator(s): 2
Innovation: 5
Approach: 8
Environment: 1

Overall Impact: This pilot randomized controlled trial (RCT) of a Mindfulness in Motion (MIM) plus Dietary Approaches to Stop Hypertension (DASH) intervention vs. a Caregiver Training control condition seeks to determine feasibility and acceptability (Aim 1); effects on caregiver stress, self-care behaviors and QOL (Aim 2); and mechanisms (role of stress reactivity/resilience in mediating effects of MIM/DASH on self-care behaviors (Aim 3) among 28 African American (AA) female Alzheimer's Disease (AD) caregivers, > 40 years old with hypertension. The project is significant, given the burden of AD caregiving and its consequences (stress, hypertension) among AA women and the lack of effective interventions for this population. The investigative team is strong with established and well described collaborations and complementary competencies in the areas needed for the successful implementation of the study: geriatrics, nutrition, health education, mind-body interventions, CV medicine, biostatistics, and neuropsychology. There is moderate novelty in the combination of a behavioral multi-component intervention (the DASH) with a mindfulness-based approach to reduce stress and improve self-care behaviors in AA caregivers of AD patients. Offsetting these strengths are several moderate to major weaknesses. First, the effects of the MIM/DASH intervention on diet, mindfulness, or stress levels observed in previous pilot studies are limited (*Geriatr Soc* 2021 Mar;69(3): 773-778). Second, the scope of the study seems too wide given its pilot nature; given its small sample size and pilot nature it is unlikely that this study can identify basic mechanisms in the adoption of healthy self-care behaviors. Third, there is limited information on the scope and design of the future RCT and on how data from the current study will inform the design for the future RCT. Fourth, there is insufficient rationale for the choice of the comparison condition – which appears to be an active control condition rather than an attention control condition. Fifth, there is minimal information on the remote platform (web? telephone?) used to deliver this complex class-based intervention; the credentials of the MIM instructors seem insufficient for the rigorous delivery of the MIM intervention and are notably inferior to those required from the Caregiver Training instructors. Lastly, the operationalization of the proposed outcomes for Aims 2 and 3 (e.g., caregiver behaviors) is unclear. Apparently, blood pressure is not an outcome of this pilot study despite the strong argument made in the significance section about the high prevalence of hypertension among African American, female AD caregivers. Overall, these weaknesses offset the strengths of this application, and its impact is thus considered moderate.

Study Timeline:

Strengths

- None noted by reviewer.

Weaknesses

- Unclear how data collection at 12 months can be implemented for all three cohorts within the limited timeline of an R21 grant.

Protections for Human Subjects:

Acceptable Risks and/or Adequate Protections

Data and Safety Monitoring Plan (Applicable for Clinical Trials Only): Acceptable

Inclusion Plans:

- Sex/Gender: Distribution justified scientifically
- Race/Ethnicity: Distribution justified scientifically
- Inclusion/Exclusion Based on Age: Distribution not justified scientifically
- Unclear why participants younger than 40 years old are excluded from the study, given that hypertension can be diagnosed at a younger age and AD caregivers can be younger.

Vertebrate Animals:

Not Applicable (No Vertebrate Animals)

Biohazards:

Not Applicable (No Biohazards)

Resource Sharing Plans:

Acceptable

Authentication of Key Biological and/or Chemical Resources:

Acceptable

Budget and Period of Support:

Recommend as Requested

THE FOLLOWING SECTIONS WERE PREPARED BY THE SCIENTIFIC REVIEW OFFICER TO SUMMARIZE THE OUTCOME OF DISCUSSIONS OF THE REVIEW COMMITTEE, OR REVIEWERS' WRITTEN CRITIQUES, ON THE FOLLOWING ISSUES:

PROTECTION OF HUMAN SUBJECTS: ACCEPTABLE

INCLUSION OF WOMEN PLAN: ACCEPTABLE

INCLUSION OF MINORITIES PLAN: ACCEPTABLE

INCLUSION ACROSS THE LIFESPAN: UNACCEPTABLE
The exclusion of individuals under the age of 40 is insufficiently justified.

COMMITTEE BUDGET RECOMMENDATIONS: The budget was recommended as requested.

Footnotes for 1 R21 AG077069-01; PI Name: Wright, Kathy Denise

+ Derived from the range of percentile values calculated for the study section that reviewed this application.

NIH has modified its policy regarding the receipt of resubmissions (amended applications).See Guide Notice NOT-OD-18-197 at https://grants.nih.gov/grants/guide/notice-files/NOT-OD-18-197.html. The impact/priority score is calculated after discussion of an application by averaging the overall scores (1-9) given by all voting reviewers on the committee and multiplying by 10. The criterion scores are submitted prior to the meeting by the individual reviewers assigned to an application, and are not discussed specifically at the review meeting or calculated into the overall impact score. Some applications also receive a percentile ranking. For details on the review process, see http://grants.nih.gov/grants/peer_review_process.htm#scoring.

MEETING ROSTER

Biobehavioral Mechanisms of Emotion, Stress and Health Study Section
Biobehavioral and Behavioral Processes Integrated Review Group
CENTER FOR SCIENTIFIC REVIEW
MESH
10/25/2021 - 10/26/2021

Notice of NIH Policy to All Applicants: Meeting rosters are provided for information purposes only. Applicant investigators and institutional officials must not communicate directly with study section members about an application before or after the review. Failure to observe this policy will create a serious breach of integrity in the peer review process, and may lead to actions outlined in NOT-OD-14-073 at https://grants.nih.gov/grants/guide/notice-files/NOT-OD-14-073.html, NOT-OD-15-106 at https://grants.nih.gov/grants/guide/notice-files/NOT-OD-15-106.html, and NOT-OD-18-115 at https://grants.nih.gov/grants/guide/notice-files/NOT-OD-18-115.html, including removal of the application from immediate review.

CHAIRPERSON(S)

MONK, CATHERINE E, PHD
PROFESSOR
DEPARTMENT OF OBSTETRICS AND GYNECOLOGY AND PSYCHIATRY
COLLEGE OF PHYSICIANS AND SURGEONS
COLUMBIA UNIVERSITY MEDICAL CENTER
NEW YORK, NY 10032

MEMBERS

BAKER, FIONA C, PHD
SENIOR PROGRAM DIRECTOR
CENTER FOR HEALTH SCIENCES
SRI INTERNATIONAL
MENLO PARK, CA 94025

BOLTZ, MARIE, PHD *
PROFESSOR
COLLEGE OF NURSING
PENN STATE UNIVERSITY
UNIVERSITY PARK, PA 16802

BRENNAN, PATRICIA A, PHD
PROFESSOR
DEPARTMENT OF PSYCHOLOGY
EMORY UNIVERSITY
ATLANTA, GA 30322

BURCH, JAMES, PHD *
PROFESSOR
DIVISION OF EPIDEMIOLOGY
DEPARTMENT OF FAMILY MEDICINE AND POPULATION HEALTH
VIRGINIA COMMONWEALTH UNIVERSITY
COLUMBIA, SC 29208

CARTER, JASON R, PHD
PROFESSOR
OFFICE OF RESEARCH, ECONOMIC DEVELOPMENT AND GRADUATE EDUCATION
DEPARTMENT OF HEALTH AND HUMAN DEVELOPMENT
MONTANA STATE UNIVERSITY
BOZEMAN, MT 59717

CASEMENT, MELYNDA D, PHD *
ASSOCIATE PROFESSOR
PSYCHOLOGY DEPARTMENT
UNIVERSITY OF OREGON
EUGENE, OR 97405

CHANG, JEN JEN, PHD *
PROFESSOR
DEPARTMENT OF EPIDEMIOLOGY
COLLEGE FOR PUBLIC HEALTH AND SOCIAL JUSTICE
ST. LOUIS UNIVERSITY
ST. LOUIS, MO 63104

CHENG, PHILIP, PHD *
ASSISTANT SCIENTIST
THOMAS ROTH SLEEP DISORDERS AND RESEARCH CENTER
HENRY FORD HEALTH SYSTEM COLUMBUS CENTER
DETROIT, MI 48202

CLOUSTON, SEAN, PHD *
ASSOCIATE PROFESSOR
PROGRAM IN PUBLIC HEALTH
STONY BROOK UNIVERSITY
STONY BROOK, NY 11794

CLUM, GRETCHEN, MA, PHD, BS *
ASSOCIATE PROFESSOR
DEPARTMENT OF GLOBAL COMMUNITY HEALTH
AND BEHAVIORAL SCIENCES
SCHOOL OF PUBLIC HEALTH AND TROPICAL MEDICINE
TULANE UNIVERSITY
NEW ORLEANS, LA 70112

FOX, RINA S, PHD *
ASSISTANT PROFESSOR
COLLEGE OF NURSING
UNIVERSITY OF ARIZONA
TUCSON, AZ 85721

GEHLERT, SARAH JANE, PHD *
PROFESSOR
DEPARTMENT OF HEALTH, ETHNICITY AND POVERTY
SUZANNE DWORAK-PECK SCHOOL OF SOCIAL WORK
UNIVERSITY OF SOUTHERN CALIFORNIA
LOS ANGELES, CA 90007

GEHRMAN, PHILIP RICHARD, PHD
ASSOCIATE PROFESSOR
DEPARTMENTS OF CLINICAL PSYCHIATRY
UNIVERSITY OF PENNSYLVANIA
PHILADELPHIA, PA 19104

GIANAROS, PETER J, PHD
PROFESSOR
DEPARTMENT OF PSYCHOLOGY
UNIVERSITY OF PITTSBURGH
PITTSBURGH, PA 15260

GISCOMBE, CHERYL L, PHD
PROFESSOR
SCHOOL OF NURSING
UNIVERSITY OF NORTH CAROLINA AT CHAPEL HILL
CHAPEL HILL, NC 27599

HOLLAND, MARGARET LANGFORD, PHD *
RESEARCH SCIENTIST
CHILD STUDY CENTER
YALE SCHOOL OF MEDICINE
YALE UNIVERSITY
NEW HAVEN, CT 06520

HONG, SUZI, PHD
PROFESSOR
HERBERT WERTHEIM SCHOOL OF PUBLIC HEALTH
PSYCHIATRY, SCHOOL OF MEDICINE
UNIVERSITY OF CALIFORNIA SAN DIEGO
LA JOLLA, CA 92093

IRISH, LEAH A, PHD *
ASSOCIATE PROFESSOR
DEPARTMENT OF PSYCHOLOGY
NORTH DAKOTA STATE UNIVERSITY
FARGO, ND 58105

LABAR, KEVIN S, PHD
PROFESSOR
DEPARTMENT OF PSYCHOLOGY AND NEUROSCIENCE
CENTER FOR COGNITIVE NEUROSCIENCE
DUKE UNIVERSITY
DURHAM, NC 27708

LARSON, CHRISTINE L, PHD
PROFESSOR
DEPARTMENT OF PSYCHOLOGY
UNIVERSITY OF WISCONSIN-MILWAUKEE
MILWAUKEE, WI 53211

LEVENDOSKY, ALYTIA A, PHD
PROFESSOR
DEPARTMENT OF PSYCHOLOGY
MICHIGAN STATE UNIVERSITY
EAST LANSING, MI 48824

MELTZER, LISA J, PHD
PROFESSOR
DEPARTMENT OF PEDIATRICS
NATIONAL JEWISH HEALTH
DENVER, CO 80206

MURROUGH, JAMES WARREN, PHD, MD
ASSOCIATE PROFESSOR
DEPARTMENT OF PSYCHIATRY AND NEUROSCIENCE
ICAHN SCHOOL OF MEDICINE AT MOUNT SINAI
NEW YORK, NY 10029

MUSTIAN, KAREN M, PHD
PROFESSOR
DEPARTMENT OF SURGERY
UNIVERSITY OF ROCHESTER SCHOOL OF MEDICINE
ROCHESTER, NY 14642

NEBLETT, ENRIQUE W JR, PHD
PROFESSOR
DEPARTMENT OF HEALTH BEHAVIOR
AND HEALTH EDUCATION
SCHOOL OF PUBLIC HEALTH
UNIVERSITY OF MICHIGAN
ANN ARBOR, MI 48109

PRATHER, ARIC ANDREW, PHD
ASSOCIATE PROFESSOR
DEPARTMENT OF PSYCHIATRY
SCHOOL OF MEDICINE
UNIVERSITY OF CALIFORNIA SAN FRANCISCO
SAN FRANCISCO, CA 94118

ROHAN, KELLY J, PHD *
PROFESSOR
DEPARTMENT OF PSYCHOLOGICAL SCIENCE
UNIVERSITY OF VERMONT
BURLINGTON, VT 05405

SALMOIRAGO-BLOTCHER, ELENA, MD, PHD
ASSOCIATE PROFESSOR
DEPARTMENT OF MEDICINE AND
AND PSYCHIATRY AND HUMAN BEHAVIOR
BROWN UNIVERSITY SCHOOL OF MEDICINE
PROVIDENCE, RI 02903

SCHERNHAMMER, EVA S, MD, DRPH *
ASSOCIATE PROFESSOR
BRIGHAM AND WOMEN'S HOSPITAL AND
HARVARD MEDICAL SCHOOL
BOSTON, MA 02115

SMITH, MICHAEL T, PHD
PROFESSOR
DEPARTMENT OF PSYCHIATRY AND BEHAVIORAL
SCIENCES
BAYVIEW MEDICAL CENTER
SCHOOL OF MEDICINE
JOHNS HOPKINS UNIVERSITY
BALTIMORE, MD 21224

SULLIVAN, ELINOR L, PHD
ASSOCIATE PROFESSOR
DEPARTMENT OF PSYCHIATRY
OREGON HEALTH AND SCIENCE UNIVERSITY
PORTLAND, OR 97239

UDDIN, MONICA, PHD
PROFESSOR
GENOMICS PROGRAM
COLLEGE OF PUBLIC HEALTH
UNIVERSITY OF SOUTH FLORIDA
TAMPA, FL 33612

VIGNATO, JULIE, PHD *
ASSISTANT PROFESSOR
COLLEGE OF NURSING
UNIVERSITY OF IOWA
IOWA CITY, IA 52242

WANG, ANDREW, MD, PHD *
ASSISTANT PROFESSOR
DEPARTMENT OF IMMUNOBIOLOGY
YALE SCHOOL OF MEDICINE
NEW HAVEN, CT 06519

WEIERICH, MARIANN R, PHD
ASSOCIATE PROFESSOR
DEPARTMENT OF PSYCHOLOGY
UNIVERSITY OF NEVADA
RENO, NV 89557

SCIENTIFIC REVIEW OFFICER

BROOKS, ALYSSA TODARO, PHD
SCIENTIFIC REVIEW OFFICER
CENTER FOR SCIENTIFIC REVIEW
NATIONAL INSTITUTES OF HEALTH
BETHESDA, MD 20892

EXTRAMURAL SUPPORT ASSISTANT

SANDERS, MARION
EXTRAMURAL SUPPORT ASSISTANT
CENTER FOR SCIENTIFIC REVIEW
NATIONAL INSTITUTES OF HEALTH
BETHESDA, MD 20892

* Temporary Member. For grant applications, temporary members may participate in the entire meeting or may review only selected applications as needed.

Consultants are required to absent themselves from the room during the review of any application if their presence would constitute or appear to constitute a conflict of interest.
